# A nosy fish

**DOI:** 10.4103/0974-2700.66538

**Published:** 2010

**Authors:** J Madana, Deeke Yolmo, S Gopalakrishnan, Sunil Kumar Saxena

**Affiliations:** Department of Otorhinolaryngology, Jawaharlal Institute of Postgraduate Medical Education and Research (JIPMER), Pondicherry - 605 006, India

Lodgment of foreign body in the nasopharynx following transoral entry is a rare entity.[1–3] Fish bones are the most common upper aerodigestive tract foreign bodies in adults, while impaction of fish in the pharynx is extremely rare.[4] This clinical record presents an unusual case of impaction of fish in the nasopharynx in an elderly male. The mode of entry, site of impaction, and the management was felt to be so interesting, hitherto unreported in the medical literature.

A 75-year-old man presented to our outpatient department of the ear, nose, throat department of Jawaharlal Institute of Postgraduate Medical Education and Research, Pondicherry, with a history of entry of fish into the mouth and its impaction in the throat 2 h earlier, while bathing in a lake. Apart from dull pain and discomfort in the throat and a degree of anxiety, the patient had no other complaints. The patient and his relatives made a few failed attempts in retrieving the fish with the hand before reaching our institute.

**Figure 1:**
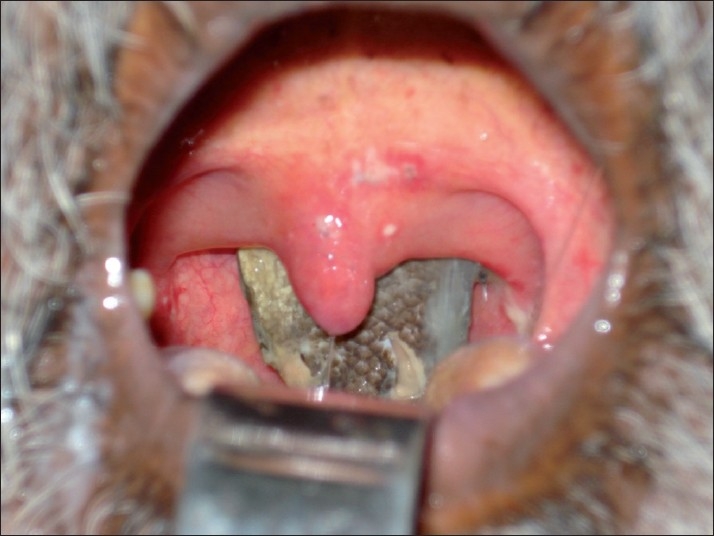
Clinical photograph showing the presence of fish with its tail and part of its body hanging in the oropharynx

**Figure 2:**
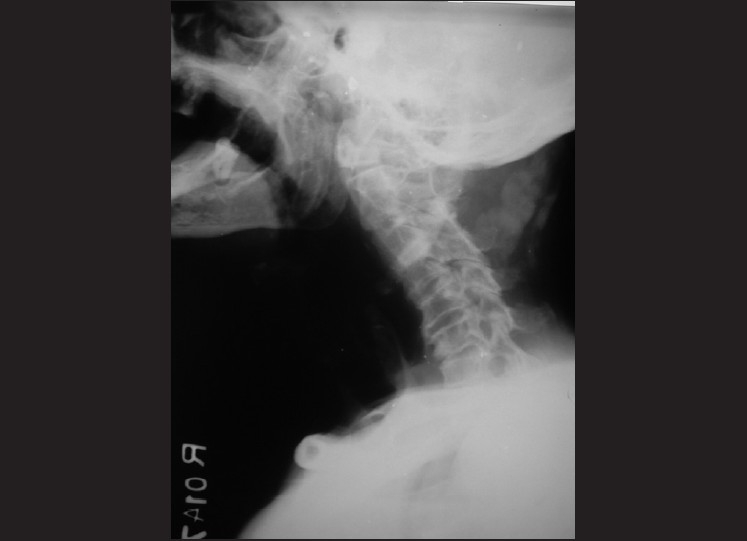
X-ray of the nasopharynx lateral view revealed the radiopaque foreign body in the naso-oropharynx

On examination, the patient was anxious but without any respiratory distress or stridor. Transoral examination revealed the presence of the fish with its tail and a part of its body hanging in the oropharynx [Figure 1]. Vital signs were normal. An immediate radiograph of the nasopharynx lateral view revealed the radiopaque foreign body in the naso-oropharynx [Figure 2].

**Figure 3:**
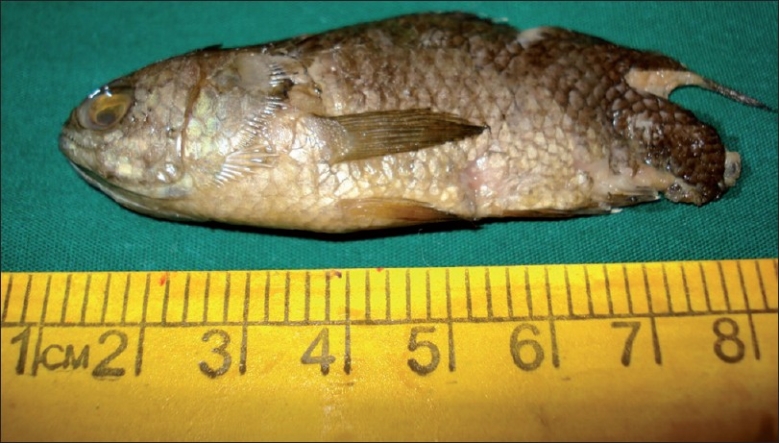
Fish in toto following transoral removal

Nasal endoscopy under local anesthesia (using 0°/4mm Karl Storz endoscope, Karl Stortz, Tutlingen, Germany) revealed the impaction of fish’s head in the nasopharynx. The impacted fish’s head was dislodged with the help of Patterson’s forceps from the nasopharynx under vision, and following throat spray with 10% lignocaine, Luc’s forceps was passed transorally to grasp its body and tail to deliver the whole of the fish gently [Figure 3]. Check endoscopy revealed a slight bruise in the posterior wall of the nasopharynx.

The postoperative period was uneventful. The patient was prescribed with antibiotics and analgesics, and discharged within 1 h of the procedure. Two weeks later, a repeat nasal endoscopy was performed and no clinically significant abnormality was detected.

Foreign bodies in the nasopharynx are extremely rare.[1,3,5] This could be due to the anatomical location of nasopharynx and nasopharyngeal isthmus, which prevent the upward migration of the foreign body after ingestion. These cases are usually attributed to forceful emesis, digital manipulation, road traffic accident, or penetrating trauma.[3]

Only 2%–4% of the inhaled foreign bodies are coughed out, while majority of them enter the esophagus or the glottis/trachea.[1] In our case, the mode of impaction of fish in the nasopharynx after entry through the oral cavity is interesting. It has been demonstrated that the sensory discrimination thresholds in the oral cavity progressively increase with increasing age,[6] which could have facilitated the oral entry in our elderly patient.

Nasopharyngeal foreign body does not usually cause symptoms, thus making the diagnosis difficult.[3] Asymptomatic foreign bodies removed after a long period of impaction have been reported.[1] Sometimes it may cause sudden respiratory distress, offensive nasal discharge, bouts of cough, unable to cry, earache, change in voice, and difficulty in swallowing.[3,5] In our case the patient was complaining of dull ache, discomfort in the throat and anxiousness, but otherwise asymptomatic.

Radiologic investigation in the form of nasopharynx X-ray, flexible nasopharyngoscopy, or rigid nasal endoscopy may be performed in cases of the inhaled/ingested foreign body as the nasopharyngeal location of the foreign body could be dangerous due to risk of fall into the larynx giving rise to sudden death.[1,3,5]

Furthermore, this clinical record highlights the importance of nasal endoscopy-assisted foreign body removal. Compared with the conventional method of nasopharyngeal foreign body removal under general anesthesia, endoscopic approach can be performed on a daycare basis under local anesthesia without hospital admission. Thus the endoscopes could play a vital role in the retrieval of nasopharyngeal foreign bodies. Moreover, this method is in accordance with the modern concept of minimally invasive surgery, thereby causing less discomfort to the patient.[5] Hence the search for the nasopharyngeal location should be attempted in cases of untraceable ingested or inhaled foreign bodies. Intravenous Conscious Sedation may be useful, which is a minimally depressed level of consciousness that retains the patient’s ability to maintain a patent airway independently and continuously and respond appropriately to physical stimulation and verbal commands.

Foreign bodies (commonly metallic ones) in the nasopharynx, although extremely rare, are usually seen in children, due to forceful emesis, digital manipulation, road traffic accident, or penetrating trauma. Fish bone impaction, while taking food has been very common. To our knowledge, this is the first report in the world literature of impaction of live fish in nasopharynx. This case indicates that transoral entry of fish and its impaction in the narrow space of nasopharynx is extremely rare, however, possible in elderly due altered sensory perception. Dislodgment of these foreign bodies, or fall onto glottis directly following transoral entry could result in fatal laryngeal obstruction. Hence, the older individuals should be extremely cautious, while taking bath in water streams.
